# Supplementary material for: Comparing Cytology Brushes for Optimal Human Nasal Epithelial Cell Collection: Implications for Airway Disease Diagnosis and Research
Source: J Pers Med. 2023 May 21;13(5):864. doi: 10.3390/jpm13050864 (PMC10222672; doi:10.3390/jpm13050864)
Supplement: Supplementary file 1 [file jpm-13-00864-s001.zip › jpm-2386954-supplementary.pdf]

Supplementary Tables

Table S1: Cell counts obtained from paediatric participants brushed under general anaesthetic for comparative assessment of the Olympus and Endoscan brushes in phase one of the study.

	**Olympus**		**Endoscan**		
	**Median**	**IQR**	**Median**	**IQR**	***p***
Total cell count ($x10^6$)	1.4	0.4 – 3.8	2.2	1.0 – 8.1	0.031
Live cell count ($x10^6$)	0.2	0.1 – 0.8	0.9	0.5 – 1.9	0.016
Cell Viability (% live)	23	8 - 48	56	44 - 61	0.097

IQR: Interquartile range

Table S2: Cell counts of participants brushed with the Endoscan brush under general anaesthetic (GA) or in the awake state in phase two of the study.

	**GA n=52**		**Awake n=93**	
	**Mean***	**95% CI**	**Mean ***	**95% CI**
Total cell count ($x10^6$)	4.9	3.9 - 6.0	1.4	1.1 - 1.8
Live cell count ($x10^6$)	1.7	1.5 - 2.1	0.4	0.3 - 0.5

*Geometric Mean

Supplementary Figure S1

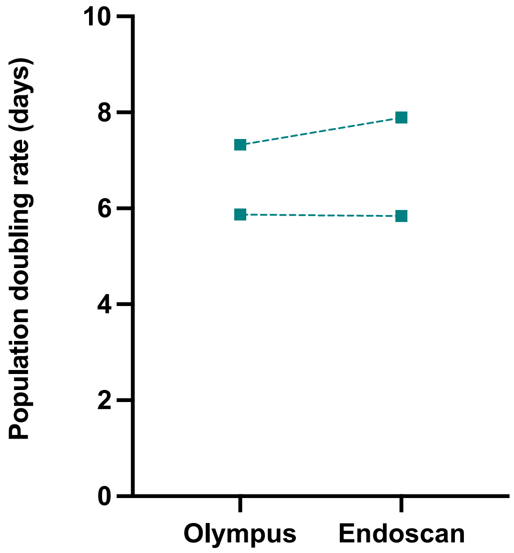

Figure S1. Population doubling rates of side-by-side cultures from the Olympus and Endoscan brush. Samples were seeded using equal cell counts from each brush. Paired samples are connected using a dashed line, n = 2.
